# Composition of False Membranes

**Published:** 1842-03

**Authors:** 


*Composition of False Membranes.*—M. Andral has clearly shown that the quantity of fibrin in the blood is increased during inflammation, and that the proportions of this substance bear a close relation to the different degrees and stages of the inflammatory affection with which it is connected. Some recent researches on the composition of false membranes, by M. Lassaigne, throw additional light on this interesting subject.

I have already (says Mr. Lassaigne) shown that the false membranes which are thrown out on mucous membranes in a high state of inflammation, are not composed of coagulated albumen, as many anatomists have supposed, but formed chiefly of a large proportion of fibrin, mixed with some soluble albumen, and moistened by a yellow colored serum, containing all the organic and inorganic elements of the blood. I have, more recently, examined the false membrane in a pig laboring under pseudo-membranous angina, and obtained the same results. The false membranes, obtained from this source, were white with a yellowish tinge, slightly elastic and extensible. On submitting them to pressure, I obtained a viscid yellowish fluid, which turned test-paper blue, and coagulated under the influence of heat and mineral acids.

A portion of the false membrane was frequently washed in cold water, to remove all the soluble matter; the residuum was a white substance, which presented all the physical and chemical characters of fibrin extracted from the blood. When digested with weak acetic acid, it became swollen, then transparent, and was entirely dissolved on the application of gentle heat. The solution, when saturated with caustic potassa, threw down white flocci, which were again dissolved by an excess of the alkali. Concentrated sulphuric, nitric, muriatic acids, and the solution of the ferro-cyanuret of potassium, threw down a white precipitate, as they do with the acetic solution of fibrin.

The water in which the false membranes were washed, was now evaporated, and there remained flocci of coagulated albumen; on continuing the evaporation to dryness, there remained a saline residuum, composed of chloride of sodium, carbonate and lactate of soda, and a little phosphate of soda, salts which exist in solution in the serum of the blood.

From these facts we may conclude, 1st. That the false membranes thrown out by serous and mucous membranes in a state of inflammation, are principally composed of the fibrin of the blood. 2. That this principle, being separated from the circulating fluid, together with a small quantity of albumen, becomes organized, and thus gives rise to the morbid products alluded to.—*Prov. Med. Jour.*, from *Journal de Chimie, June* 1840.

